# Supplementary figures and images for: HTR6 and SSTR3 ciliary targeting relies on both IC3 loops and C-terminal tails
Source: Life Sci Alliance. 2020 Dec 28;4(3):e202000746. doi: 10.26508/lsa.202000746 (PMC7772773; doi:10.26508/lsa.202000746)

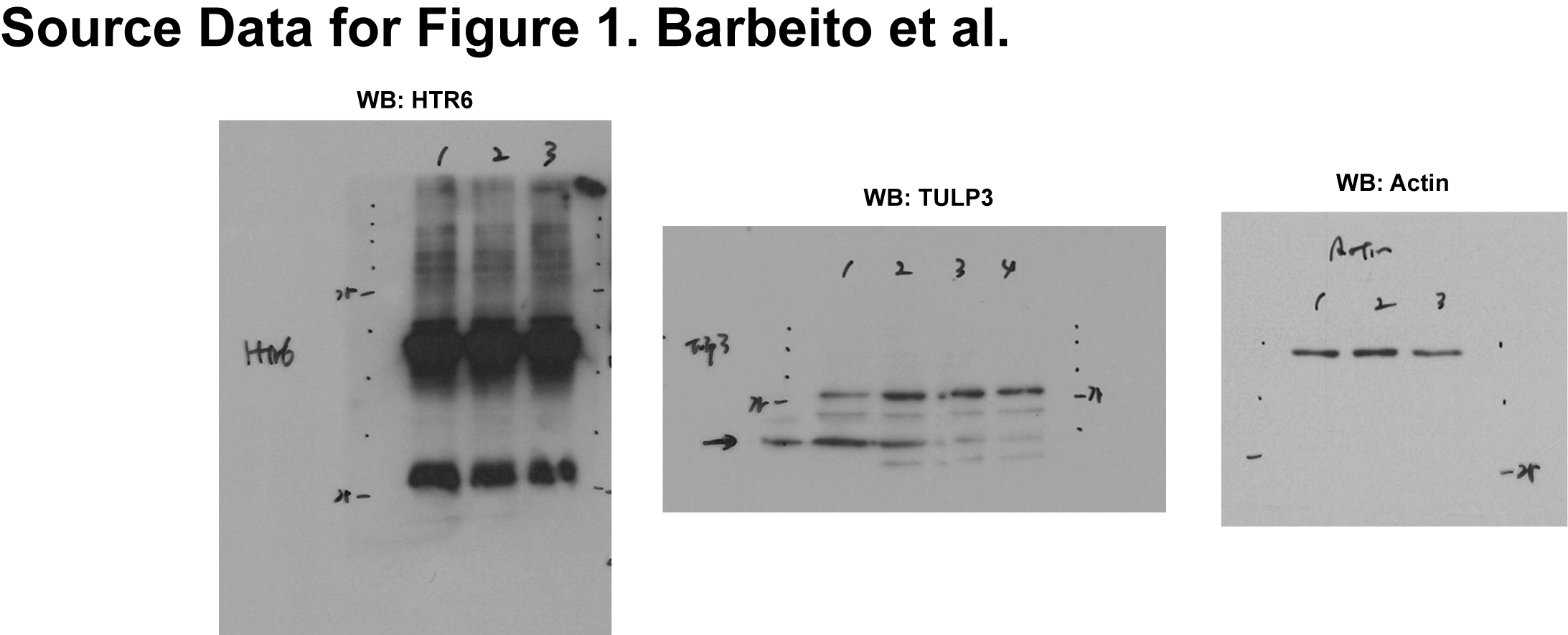

Supplement: Supplementary file 1 [file LSA-2020-00746_SdataF1.tif]

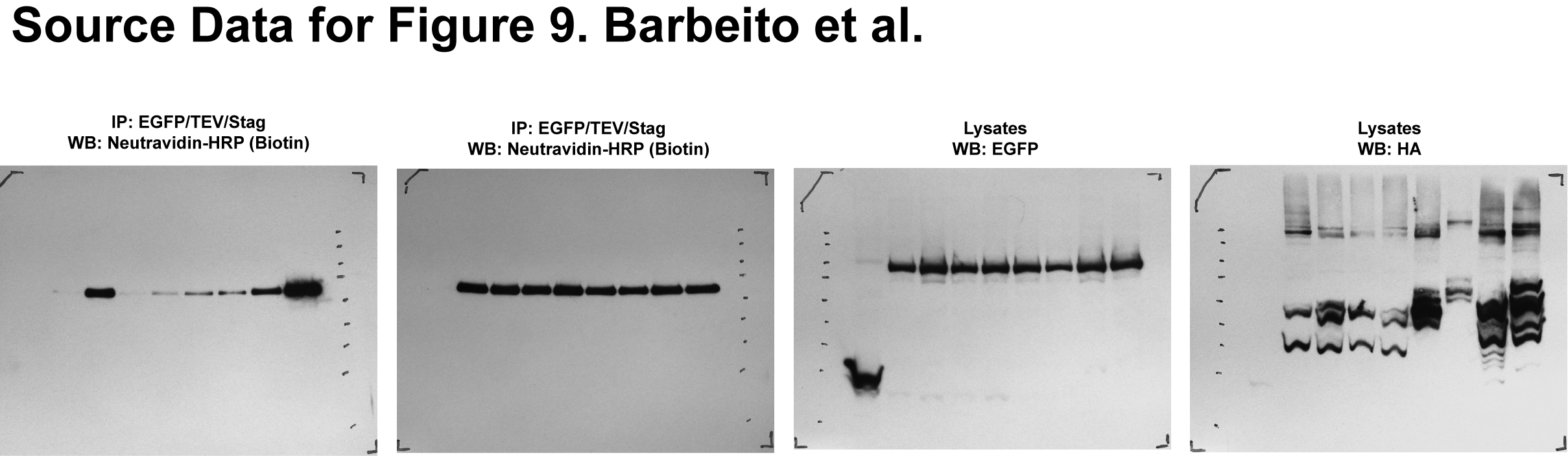

Supplement: Supplementary file 2 [file LSA-2020-00746_SdataF9.tif]

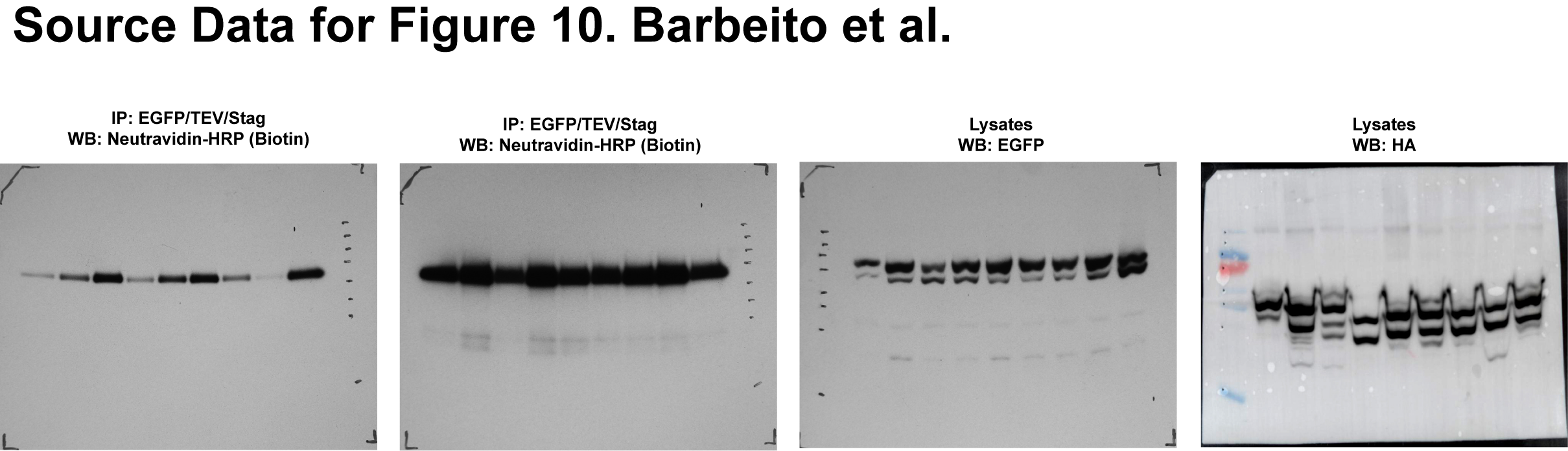

Supplement: Supplementary file 3 [file LSA-2020-00746_SdataF10.tif]

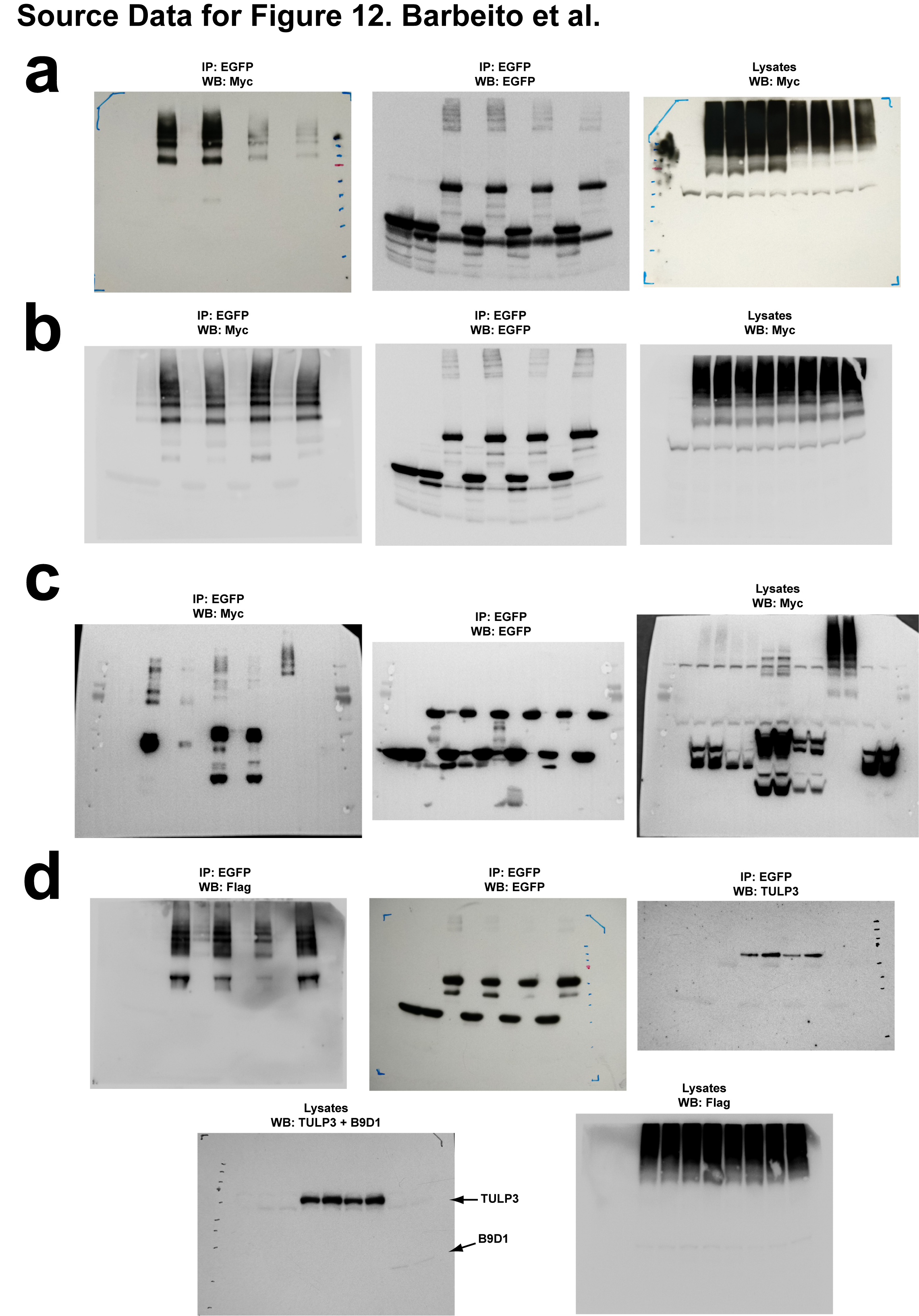

Supplement: Supplementary file 4 [file LSA-2020-00746_SdataF12.tif]
